# A Multidisciplinary Approach to a Treatment-Resistant Anti-N-Methyl-D-Aspartate Receptor Encephalitis Patient in the ICU: A Case Report

**DOI:** 10.7759/cureus.85605

**Published:** 2025-06-09

**Authors:** Zaza Aladashvili, Bianca Lupia, Thalia B Rodriguez, Amalia B Pontes, Yaroslav Buryk

**Affiliations:** 1 Internal Medicine/Critical Care Medicine, Jackson Memorial Hospital, Miami, USA; 2 Faculty of Medicine, Tbilisi State Medical University, Tbilisi, GEO; 3 Internal Medicine, St. George's University School of Medicine, St. George's, GRD; 4 Medicine, Ross University School of Medicine, Miramar, USA; 5 Internal Medicine, Jackson Memorial Hospital, Miami, USA; 6 Pulmonary and Critical Care Medicine, Jackson Memorial Hospital, Miami, USA; 7 Pulmonary and Critical Care Medicine, University of Miami Miller School of Medicine, Miami, USA

**Keywords:** anti-nmdar encephalitis, ivig treatment, neurology and critical care, ovarian teratoma, pulmonary critical care, rituximab therapy

## Abstract

Anti-N-methyl-D-aspartate receptor (anti-NMDAR) encephalitis is an autoimmune disorder characterized by complex neuropsychiatric features and the presence of immunoglobulin G (IgG) antibodies against the receptor subunit 1 (NR1) of the NMDA receptors in the central nervous system. In this case report, we present a 31-year-old female patient who was admitted to the hospital with altered mental status, hallucinations, and seizure activity. After the failure of first-line treatment with intravenous immunoglobulin/plasma exchange (IVIG/PLEX) started at the previous facility, the patient was started on a trial of rituximab, which showed clinical improvement. This case report aims to shed light on the progression of a rare disorder, early tumor detection, individualized immunotherapy, and a multidisciplinary approach to treatment plans.

## Introduction

Anti-N-methyl-D-aspartate receptor (anti-NMDAR) encephalitis is a rare autoimmune condition; however, it is one of the most common causes of encephalitis [1]. It is characterized by anti-glutamate receptor subunit epsilon 1 (anti-GluN1) autoantibodies attacking the N-methyl-D-aspartate (NMDA) receptors in the cerebrospinal fluid (CSF). Anti-N-methyl-D-aspartate (anti-NMDA) encephalitis is classified as a neuropsychiatric disorder with additional symptoms such as central hypoventilation, impaired consciousness, loss of memory, and seizures [2]. This rare disorder has an incidence of one to five cases per million individuals each year [2]. A lack of awareness regarding this condition leads to many misdiagnoses, which may contribute to the low incidence [3]. Anti-NMDAR encephalitis primarily affects women, with a greater incidence in young adults and children, commonly associated with ovarian teratomas [2]. 

The mechanism of anti-NMDAR encephalitis remains unclear, although authors propose that the pathophysiology is associated with ovarian teratomas, which express and expose N-methyl-D-aspartate receptors to the immune system, causing the production of antibodies. In another case, plasma cells and B-cells forming anti-glutamate receptor subunit epsilon 1 antibodies evade immune regulators. Another hypothesis suggests that anti-NMDAR encephalitis is associated with Toxoplasma gondii or herpes simplex virus [4,5]. 

Anti-NMDAR encephalitis has a multifaceted presentation that differs in everyone. The disease often commences with a prodromal phase of flu-like symptoms lasting five to fourteen days. Psychiatric manifestations, such as paranoia, hallucinations, and cognitive impairment, follow the prodromal phase. Next is the unresponsive phase, in which patients fail to obey verbal commands and respond to pain or visual stimuli. The latter stages of the disease include severe hyperkinetic and neurological disturbances, such as altered consciousness, seizures, dysautonomia, and dyskinesias [6]. Upon tumor treatment via removal or immunosuppressive therapy, patients may reach a gradual recovery phase [4,7]. 

The gold standard for diagnosis is CSF analysis that reveals anti-NMDAR antibodies. Electroencephalogram (EEG) and magnetic resonance imaging (MRI) results further support the diagnosis of electrical disturbances and brain inflammation, such as disorganized or slow activity and delta brush. Indications for diagnosis of anti-NMDAR encephalitis rely on the abrupt onset (within 12 weeks) of four or more of the following symptoms: cognitive impairment or unusual behavior, unusual speech, movement disorders, seizures, autonomic dysfunction, and reduced consciousness [2,7]. Diagnosis is also probable if three of these symptoms and a teratoma are present. Prognosis greatly depends on early diagnosis and immunotherapy, such as intravenous immunoglobulin (IVIG), plasmapheresis, or corticosteroids. If there is tumor involvement, surgical resection is imminent to ensure complete recovery [2]. Supportive care with anticonvulsants, mechanical ventilation, nutritional intake, and antipsychotics effectively manages symptoms [7]. 

This report presents a rare case of anti-NMDAR encephalitis in a young female patient, highlighting the necessity for early detection, prompt intervention, and multidisciplinary care in addressing this complicated medical condition. The discussion emphasizes the challenges associated with diagnosis, therapeutic interventions, and prognostic outcomes.

## Case presentation

A 31-year-old woman diagnosed with anti-NMDAR encephalitis, initially admitted to a different facility in November 2024, presented with symptoms of altered mental status, seizures, and hallucinations. A lumbar puncture was performed, confirming the diagnosis of anti-NMDAR encephalitis. First-line treatment with IVIG and plasma exchange (PLEX) was initiated, along with percutaneous endoscopic gastrostomy and tracheostomy tube placement. The obstetrics and gynecology (OB-GYN) team ordered a magnetic resonance imaging (MRI) scan to evaluate the patient's ovaries and identified a 3.5 cm left ovarian mass. The patient underwent a left salpingo-oophorectomy and biopsy of the mass, which confirmed an ovarian teratoma, a tumor commonly associated with anti-NMDAR encephalitis. Despite the surgery, the patient showed no clinical improvement, and her condition worsened over the following month. Persistent agitation, movement disorders, and autonomic instability suggested treatment-resistant disease, prompting the family to request a transfer to our facility.

Upon arrival in February 2025 (day 0), the patient was admitted to the Pulmonary Intensive Care Unit (PICU) due to acute hypoxic respiratory failure and full ventilator dependence. A transvaginal ultrasound revealed a 2 cm solid right adnexal mass. The OB-GYN team deferred a repeat MRI due to the patient’s worsening respiratory status despite normal cardiovascular function. On physical examination, the patient demonstrated orofacial dyskinesia and spontaneous extremity movements. The treatment goal was to gradually modify the existing multi-drug regimen.

Because first-line IVIG and PLEX therapy had failed to show clinical benefit, the medical team initiated rituximab, a second-line immunotherapy. The full medication regimen administered during PICU admission is presented in Table 1. Rituximab is known to improve clinical outcomes in treatment-resistant anti-NMDAR encephalitis. With family consent, the first dose was administered upon admission (day 0), and a second dose followed two weeks later (day 14). No significant clinical change was observed between doses. However, the addition of antiepileptic and neuroleptic medications after the second dose led to mild stabilization characterized by improved alertness, increased command-following ability, reduced orolingual dyskinesia, and slight improvement in spontaneous extremity movements. The patient's oxygen saturation on mechanical ventilation remained >92%; renal function was stable, and glucose was maintained below 180 mg/dL. A week following the second rituximab dose (around day 21), the patient transitioned from assisted control to synchronized intermittent mandatory ventilation. Despite clinical improvement, she remained in the PICU. A third rituximab dose was scheduled six months later. Venous thromboembolism and peptic ulcer prophylaxis were continued throughout hospitalization.

**Table 1:** Medication Comparison Table: Admission vs Long-Term Acute Care Transfer *Continued Medications:* Clobazam, clonidine, diazepam, docusate sodium, famotidine, gabapentin, heparin, lacosamide, levetiracetam, polyethylene glycol (PEG/MiraLAX), propranolol, sennosides, acetaminophen (PRN), and midazolam (PRN) Dose/Frequency Adjustments: Gabapentin: TID → QID Heparin: TID → Q8H Clonidine: TID → BID Oxycodone: 10 mg → 5 mg *New Medications at Transfer:* Aripiprazole and olanzapine (PRN) *Discontinued at Transfer:* Antibiotics: Ampicillin-Sulbactam, meropenem, and sulfamethoxazole-trimethoprim (SMX-TMP) ICU/Short-term: Dexmedetomidine (Precedex) drip, rituximab, and diphenhydramine Others: Bromocriptine and oxycodone IR (PRN) FT: Feeding tube; IVPB: Intravenous piggyback; Q4H, Q6H, Q8H, etc. – Every 4, 6, or 8 hours; PRN: As needed; IM: Intramuscular; SC: Subcutaneous; IV: Intravenous; BID: Twice daily; TID: Three times daily; QID: Four times daily; SIMV: Synchronized intermittent mandatory ventilation; AC: Assist control; ICU: Intensive care unit; VTE: Venous thromboembolism; PEG: Polyethylene glycol (MiraLAX)

Medication Name	At Admission	At Transfer to a Long-Term Acute Care Facility
Acetaminophen	650 mg/20.3 mL, FT, ONCE	650 mg, FT, Q6H (PRN)
Ampicillin-Sulbactam	3 g, IVPB, Q4H	—
Aripiprazole	—	20 mg, ORAL, DAILY
Bromocriptine	5 mg, FT, BID	—
Clobazam	5 mg, FT, BID	5 mg, FT, BID
Clonidine	0.3 mg, FT, TID	0.3 mg, FT, BID
Diazepam	5 mg, FT, TID	5 mg, FT, TID
Diphenhydramine	50 mg, IV PUSH, ONCE	—
Docusate Sodium	100 mg, FT, BID	100 mg, FT, BID
Famotidine	20 mg, FT, BID	20 mg, FT, BID
Fluarix Trivalent (Flu Vaccine)	0.5 mL, IM, BEFORE DISCHARGE	0.5 mL, IM, BEFORE DISCHARGE
Gabapentin	300 mg, FT, TID	300 mg, FT, QID
Heparin	5,000 units, SC, TID	5,000 units, SC, Q8H
Lacosamide	200 mg, FT, BID	200 mg, FT, BID
Levetiracetam	1,000 mg, FT, Q12H	1,000 mg, FT, Q12H
Meropenem	1,000 mg, IVPB, Q8H	—
Oxycodone IR (Scheduled)	10 mg, FT, Q4H	5 mg, FT, Q4H
Oxycodone IR (PRN)	10 mg, FT, Q4H	—
PEG (MiraLAX)	17 g, FT, DAILY	17 g, FT, DAILY
Propranolol	20 mg, FT, TID	20 mg, FT, TID
Rituximab	1,000 mg, IVPB, ONCE	—
Sennosides	17.6 mg, FT, BID	17.6 mg, FT, BID
SMX-TMP (Bactrim)	320 mg, FT, BID	—
Dexmedetomidine (Drip)	400 mcg/100 mL, IV DRIP, 2.5 mL/hr	—
Fentanyl (PRN)	50 mcg, IV, Q2H	50 mcg, IM PUSH, Q2H
Midazolam (PRN)	2 mg, IV PUSH, Q2H	2 mg, IV PUSH, Q2H
Olanzapine (PRN)	—	5 mg, IV, ONCE

The patient spent 30 days in our facility, during which the first dose of rituximab was given on the day of admission, and the second dose was administered two weeks later. After the second dose, the patient remained in our care for an additional two weeks. This multidisciplinary approach led to improved alertness and command-following ability, allowing sedative reduction. Motor function also improved, particularly leg extension against resistance, though orolingual dyskinesia persisted. The patient was transferred to a long-term acute care facility. Discharge recommendations included OB-GYN consultation for the right adnexal mass and continuation of the adjusted drug regimen with anti-seizure medications. A pelvic MRI was attempted but was inconclusive due to motion artifact. The exact nature of the second right adnexal mass remains uncertain; differential diagnoses include a second teratoma or hemorrhagic ovarian cyst. Follow-up imaging or biopsy could not be performed due to the patient’s clinical instability, which influenced ongoing management decisions. The long-term plan includes sedative weaning and initiation of physical therapy, occupational therapy, and speech therapy.

## Discussion

Anti-NMDAR encephalitis classically presents with neuropsychiatric symptoms such as seizures, dyskinesia, and autonomic instability. These symptoms can mimic other psychiatric diseases, leading to delays in diagnosis and treatment. A cohort study showed that patients who had a delayed diagnosis ended up staying longer in the intensive care unit (ICU), had a slower recovery, and had a higher risk of long-term disability [8]. In contrast, when detected earlier, there were better patient outcomes, faster recoveries, and lower relapse rates [8], emphasizing the importance of early detection to establish care sooner.

In our patient, the discovery of potential bilateral ovarian teratomas, initially a left-sided mass followed by a contralateral adnexal mass, represents a rare but clinically significant finding. The presence of bilateral teratomas is an uncommon finding in women with anti-NMDAR encephalitis, as it only occurs in about 11% of cases [9]. The second adnexal mass was discovered after the patient showed no significant improvement post-oophorectomy and first-line immunotherapy, prompting repeat imaging. While a definitive diagnosis of the second mass was not confirmed histologically, it raised strong suspicion for a synchronous or metachronous teratoma. This highlights the importance of repeated and thorough pelvic imaging in patients who fail to improve following tumor resection and first-line immunotherapy. In such cases, failure of clinical response may itself serve as an indication to reassess for residual or contralateral neoplastic sources, even in the absence of classic imaging findings.

The pathophysiological link between ovarian teratomas and anti-NMDAR encephalitis is thought to stem from ectopic expression of NMDA receptors by neural tissue within the teratoma. This molecular mimicry can lead to a breakdown of immune tolerance, triggering B-cell activation and the production of pathogenic antibodies against NMDA receptors in the central nervous system. Removal of the teratoma often leads to symptom improvement; however, as our case illustrates, clinical improvement may still require additional immunomodulation, particularly when another suspected mass is not surgically removed.

When first-line immunotherapy fails, second-line treatment with rituximab, a monoclonal anti-CD20 antibody, is typically considered. Rituximab depletes B-lymphocytes, thereby reducing autoantibody production [8]. In an observational cohort study with over 500 patients, those who had received rituximab and/or cyclophosphamide showed improved long-term outcomes, faster recoveries, and were less likely to relapse compared to patients treated only with first-line therapy [8], supporting the use of these agents in refractory cases. In our case, cyclophosphamide was not initiated due to its potential gonadotoxicity and the patient's age and reproductive considerations. Rituximab was selected through a multidisciplinary consensus as the safer immunosuppressive option for this patient.

Our patient showed clinical improvement after rituximab administration despite the second adnexal mass not being surgically addressed. This raises the possibility that the persistent symptoms were more responsive to immunotherapy than to further oncologic management or that the contralateral mass was not immunologically active. While this cannot be definitively resolved without histopathologic confirmation, it reinforces the utility of second-line immunosuppression in managing persistent symptoms, even in the setting of an untreated mass.

Anti-NMDAR encephalitis, though rare, should be included in the differential diagnoses for any patient with altered mental status or sudden psychiatric changes. Particular attention should be given to young women with subacute psychosis or seizures, especially when initial treatments fail, and even when one tumor has already been identified and resected. This is particularly important in cases presenting with new-onset psychosis, as studies suggest that up to 10% of patients experiencing first-episode psychosis may exhibit underlying signs of autoimmune encephalitis [10]. Emerging evidence supports that persistent or worsening symptoms, despite adequate initial management, should trigger reconsideration of the underlying etiology, including the possibility of bilateral tumors or incomplete immunosuppression.

Coordinated, multidisciplinary care, including neurology, critical care, OB-GYN, and psychiatry, is essential for optimizing outcomes in severe presentations, as demonstrated in our patient. This case further emphasizes the importance of flexible, persistent, and individualized treatment strategies, and the growing need to formalize comprehensive guidelines for managing refractory anti-NMDAR encephalitis.

## Conclusions

This case highlights the importance of promptly diagnosing anti-NMDAR encephalitis, a condition that often presents with a wide range of neurological and psychiatric symptoms. Multiple medical departments collaborated in managing the patient, resulting in modest improvements, specifically in motor function and arousal. However, the patient remained agitated and continued to experience orolingual dyskinesia despite ongoing treatments.

Comprehensive oncological evaluation is essential in all female patients with anti-NMDAR encephalitis, given the strong association with ovarian teratomas, and repeated imaging may be warranted in refractory cases to detect synchronous or metachronous tumors. This case reinforces the necessity for continued research in this field and illustrates the importance of maintaining flexible and adaptive treatment strategies in managing complex and sensitive conditions. Furthermore, early and aggressive second-line immunotherapy, such as rituximab, has been shown to improve long-term outcomes and should be considered promptly in patients who fail to respond to first-line therapies.
